# Components of Mineralocorticoid Receptor System in Human DRG Neurons Co-Expressing Pain-Signaling Molecules: Implications for Nociception

**DOI:** 10.3390/cells14151142

**Published:** 2025-07-24

**Authors:** Shaaban A. Mousa, Xueqi Hong, Elsayed Y. Metwally, Sascha Tafelski, Jan David Wandrey, Jörg Piontek, Sascha Treskatsch, Michael Schäfer, Mohammed Shaqura

**Affiliations:** 1 Department of Anesthesiology and Intensive Care Medicine, Charité Campus Benjamin Franklin, Charité—Universitätsmedizin Berlin, Corporate Member of Freie Universität Berlin, Humboldt-Universität zu Berlin, and Berlin Institute of Health, Hindenburgdamm 30, 12203 Berlin, Germany; xueqi.hong@charite.de (X.H.); micha.schaefer@charite.de (M.S.);; 2 Department of Anesthesiology and Intensive Care Medicine, Charité Campus Mitte and Campus Virchow Clinic, Charité—Universitätsmedizin Berlin, Corporate Member of Freie Universität Berlin, Humboldt-Universität zu Berlin, and Berlin Institute of Health, Charitéplatz 1, 10117 Berlin, Germanyjan.wandrey@charite.de (J.D.W.); 3 BIH Charité Digital Clinician Scientist Program, BIH Biomedical Innovation Academy, Berlin Institute of Health at Charité—Universitätsmedizin Berlin, Charitéplatz 1, 10117 Berlin, Germany; 4 Clinical Physiology/Nutritional Medicine, Department of Gastroenterology, Rheumatology and Infectious Diseases, Charité—Universitätsmedizin Berlin, 12203 Berlin, Germany

**Keywords:** corticosteroid receptors, aldosterone synthase, pain signaling molecules, peripheral sensory neurons, pain, human, rats

## Abstract

The mineralocorticoid receptor (MR), traditionally associated with renal function, has also been identified in various extrarenal tissues, including the heart, brain, and dorsal root ganglion (DRG) neurons in rodents. Previous studies suggest a role for the MR in modulating peripheral nociception, with MR activation in rat DRG neurons by its endogenous ligand, aldosterone. This study aimed to determine whether MR, its protective enzyme 11β-hydroxysteroid dehydrogenase type 2 (11β-HSD2), its endogenous ligand aldosterone, and the aldosterone-synthesizing enzyme CYP11B2 are expressed in human DRG neurons and whether they colocalize with key pain-associated signaling molecules as potential targets for genomic regulation. To this end, we performed mRNA transcript profiling and immunofluorescence confocal microscopy on human and rat DRG tissues. We detected mRNA transcripts for MR, 11β-HSD2, and CYP11B2 in human DRG, alongside transcripts for key thermosensitive and nociceptive markers such as TRPV1, the TTX-resistant sodium channel Nav1.8, and the neuropeptides CGRP and substance P (Tac1). Immunofluorescence analysis revealed substantial colocalization of MR with 11β-HSD2 and CGRP, a marker of unmyelinated C-fibers and thinly myelinated Aδ-fibers, in human DRG. MR immunoreactivity was primarily restricted to small- and medium-diameter neurons, with lower expression in large neurons (>70 µm). Similarly, aldosterone colocalized with CYP11B2 and MR with nociceptive markers including TRPV1, Nav1.8, and TrkA in human DRG. Importantly, functional studies demonstrated that prolonged intrathecal inhibition of aldosterone synthesis within rat DRG neurons, using an aldosterone synthase inhibitor significantly downregulated pain-associated molecules and led to sustained attenuation of inflammation-induced hyperalgesia. Together, these findings identify a conserved peripheral MR signaling axis in humans and highlight its potential as a novel target for pain modulation therapies.

## 1. Introduction

Several years ago, glucocorticoid and mineralocorticoid receptors were localized to neurons of the central nervous system using radioligand binding techniques [1,2]. These early findings laid the groundwork for later studies implicating corticosteroid receptors in the regulation of sensory neuropeptides such as CGRP [3] and substance P [4] in peripheral sensory neurons. Based on this evidence, functional studies demonstrated that local application of the MR antagonist eplerenone significantly attenuated nociceptive behavior in rats [5]. Subsequent work identified MR expression predominantly in nociceptive fibers, namely unmyelinated C-fibers and thinly myelinated Aδ-fibers [6]. Consistent with these findings, intraplantar administration of MR agonists enhanced, while MR antagonists reduced mechanical nociceptive sensitivity [7]. Electrophysiological studies further confirmed that MR activation increased, and MR inhibition decreased the excitability of peripheral sensory neurons in the presence of inflammation [5], suggesting the putative involvement of an intrinsic, endogenous MR signalling mechanism. This notion was corroborated by the discovery of the aldosterone-synthesizing enzyme CYP11B2 and its product, aldosterone, within peripheral sensory neurons [7,8]. Given that MR is a member of the nuclear hormone receptor family, typically exerting its effects via genomic regulation, recent findings suggest MR signalling may extend beyond the modulation of sensory neuropeptides and might also target other key pain signalling molecules in peripheral sensory neurons, which finally might have an impact on mechanical nociception [8]. Accordingly, the current study investigates whether MR, 11β-HSD2, aldosterone, and CYP11B2 are expressed in human DRG neurons and whether they colocalize with key nociceptive signalling molecules. We also present preliminary functional evidence from a rodent model demonstrating that sustained intrathecal inhibition of aldosterone synthesis results in downregulation of pain-associated markers and prolonged attenuation of hyperalgesia. These findings aim to bridge rodent and human data, supporting the translational potential of targeting peripheral MR signalling in pain management.

## 2. Materials and Methods

### 2.1. Collection of Human and Rat DRG Tissue Samples

Frozen human DRGs (L4) from two young fatal accident victims at ages 20 and 22 years (one female, 20 years old, and one male, 22 years old) were obtained from AnaBios Corporation and Donor Network West (San Ramon, CA, USA). Upon collection, DRG tissue samples were immediately preserved, either in liquid nitrogen or fixed in 4% paraformaldehyde prior to shipment. The frozen tissue DRG samples were divided in half to obtain two technical replicates and used for quantitative real-time PCR (see below for more details). The suppliers ensured that informed consent was obtained from all donors contributing biological materials, along with all other necessary authorizations, consents, and permissions required for the transfer and use of the materials for research purposes. This study was conducted in accordance with the Declaration of Helsinki and was approved by the Institutional Review Board of the Charité (EA1/228/14). Additionally, approval for animal experiments was obtained from the competent authority in Berlin (LaGeSo; approval number G0169/19).

### 2.2. Experimental Animal Protocols

Wistar rats (250–300 g; Janvier, Berlin, Germany) (*n* = 5–6) were briefly anesthetized with isoflurane and received an intraplantar (i.pl.) injection of 0.15 mL Freund’s Complete Adjuvant (FCA; Calbiochem, Darmstadt, Germany) into the right hind paw. This procedure reliably induces localized inflammation, characterized by increased mechanical sensitivity. In this model of FCA-induced hindpaw inflammation, the effects of continuous intrathecal (i.t.) administration of the aldosterone synthase inhibitor FAD286 or vehicle were evaluated. Outcome measures included mechanical paw pressure thresholds (PPTs), DRG aldosterone content (via ELISA), and DRG gene expression levels of mineralocorticoid receptor (MR), 11β-HSD2, CYP11B2, and pain-related signaling molecules.

For i.t. drug delivery, Alzet osmotic minipumps (volume: 2000 μL; flow rate: 5 μL/h) were filled with 0.9% NaCl, either with or without FAD286 (0.3 μg/μL), and connected to an i.t. catheter. The solution was continuously infused over a 4-day period. Intrathecal catheter implantation was performed as previously described [8,9]. Briefly, a needle was inserted at a 30° angle between the L4 and L5 vertebrae to access the intrathecal space. Correct placement was confirmed by reflexive tail or hindlimb movement, indicating dura penetration. A PE-10 catheter (connected to PE-60 tubing; Portex Ltd., Hythe, Kent, UK) was then positioned and attached to the pump. After 4 days of FCA-induced inflammation with or without FAD286 treatment, L3–L5 DRGs were harvested. Tissues were either fresh-frozen for qRT-PCR analysis or fixed in formalin for subsequent immunohistochemical analysis.

### 2.3. Quantitative RT-PCR in Human and Rat DRG

Total RNA was extracted from human and rat DRGs and reverse transcribed into cDNA using an RNeasy (Qiagen, Hilden, Germany) and cDNA synthesis kit (Takara, Göteborg, Sweden), respectively, as previously described [10]. The following are specific primer pairs for MR, 11ß-HSD2, CYP11B2, TRPV1, TRPV2, TRPV4, TRPA1, TRPM8, Nav.1.8, Nav1.9, CGRP, Tac1, and 18S (see Supplementary Table S1). TaqMan quantitative polymerase chain reaction was performed with a SYBR^®^ Green master mix following the manufacturer’s instructions (Thermo Fisher Scientific, Hennigsdorf, Germany). Amplification was carried out for 40 cycles, each consisting of 15 s at 95 °C, for all genes except 8S of 30 s at 60 °C. A temperature just below the specific melting temperature was employed for detection of fluorescence-specific products (GR: Tm 83 °C, 18S: Tm 88 °C). Each human or rat tissue sample was measured in triplicate and the evaluation was performed according to the ΔΔCT method, i.e., ΔCt values were obtained by the Ctgene–Ct18S housekeeping gene and sub-sequently related to ΔCt values of MR [11].

### 2.4. Quantification of Aldosterone in DRG

To quantify aldosterone levels in the DRG of FCA-treated rats with (FAD286 treatment) or without (vehicle) continuous i.th. administration of the aldosterone synthase inhibitor, a commercial ELISA kit (R&D Systems, cat.# KGE016, Minneapolis, MN, USA) was used in accordance with the manufacturer’s instructions, also as outlined by [12]. Each measurement was performed in triplicate. Optical density (O.D.) readings were obtained at 450 nm using a microplate reader (LS45, Perkin Elmer, Waltham, MA, USA). For each standard, control, and sample, the mean of the triplicate readings was calculated after subtracting the average non-specific binding (NSB) O.D. values. A standard curve was then generated using a four-parameter logistic (4-PL) model with the accompanying software (LS45, Perkin Elmer, Waltham, MA, USA).

### 2.5. Immunohistochemistry in Human and Rat DRG

All tissue preparations were performed according to the protocols described in previous studies [10,13]. Fixed tissues from both human and rat sources were cryoprotected overnight at 4 °C in PBS solution containing 10% sucrose. Following this step, the tissues were rinsed in PBS and stored at –80 °C to maintain cryoprotection. Immunostaining was performed on every fourth section of serially cut DRGs (10 μm) from each rat (*n* = 5) and human donor (*n* = 2), with a minimum of 10 sections stained per antibody.

DRG tissue sections (10 µm thick), mounted on slides, were first washed in PBS and subsequently treated with freshly prepared ice-cold sodium borohydride (1 mg/mL in PBS) for three intervals of 10 min each before proceeding to the blocking phase. Blocking was carried out for 60 min using a solution of PBS supplemented with 0.3% Triton X-100, 1% bovine serum albumin (BSA), and 10% each of goat and donkey serum. Sections were then incubated overnight with primary antibodies targeting MR, 11ß-HSD2, CYP11B2, CGRP, TRPV1, Nav1.8, and TrkA, either individually or in combination, for double immunofluorescence (see Supplementary Table S2). The next day, slides were rinsed in PBS and incubated with Alexa Fluor 594-conjugated donkey anti-rabbit or anti-mouse antibodies (Vector Laboratories), along with Alexa Fluor 488-conjugated goat anti-guinea pig or anti-mouse antibodies (Thermo Fisher Scientific, Hennigsdorf, Germany). Nuclei were counterstained using DAPI (0.1 µg/mL in PBS; Sigma), which produces a bright blue fluorescence. After final PBS washes, the sections were mounted in Vectashield (Vector Laboratories) and examined under a confocal laser scanning microscope (LSM510; Carl Zeiss, Göttingen, Germany), equipped with multiple lasers (argon: 458/488/514 nm; green HeNe: 543 nm; red HeNe: 633 nm), as previously described [13]. To ensure consistency across image sets, identical parameters were used for brightness, contrast, pinhole size, and scanning duration. Fluorescence signals were captured using a PLAN-NEOFLUAR 40×/1.3 oil immersion objective (Zeiss, Oberkochen, Germany). Quantitative analysis of MR and aldosterone/CGRP immunoreactivity across varying neuronal diameters in human and rat DRG sections was conducted by blinded evaluator using the Zeiss Zen 2009 software (Carl Zeiss MicroImaging GmbH, Göttingen, Germany) [7]. Values were presented as means ± SD.

To validate staining specificity, controls were included: tissue sections processed without primary or secondary antibodies (to assess background autofluorescence), and sections incubated only with secondary antibodies (to detect non-specific binding), in accordance with previous studies (see Supplemental Figure S1). Our mouse monoclonal antibody against the MR (private gift from Prof. Elise Gomez-Sanchez, Jackson, MS, USA) has previously been shown in rat [14,15,16,17] and human [10,18] studies to be highly specific; the specificity of the purified polyclonal rabbit anti-aldosterone (Novus Biologicals, Wiesbaden, Germany) was confirmed by our previous immunoprecipitation for aldosterone and subsequent demonstration of the mineralocorticoid receptor by Western blot [12].

### 2.6. ELISA of DRG Aldosterone Content

DRG (*n* = 5) were placed in sterile Dulbecco’s modified Eagle’s medium at 4 °C and enzymatically digested with collagenase type 2 (37 °C, 50 min) followed by 0.025% trypsin (37 °C, 10 min), then mechanically dissociated and centrifuged at 500× *g* and 300× *g* for 5 min each. Cells were cultured overnight in minimum essential medium with 10% horse serum and 50 μg/mL penicillin/streptomycin in 24-well plates (1.9 cm^2^) at 37 °C with 5% CO_2_. Aldosterone levels were measured using a commercial ELISA kit (KGE016, R&D Systems) per the manufacturer’s protocol and prior methods. Assays were conducted in triplicate from 5 rats. Optical density at 450 nm was measured with a microplate reader (LS45, Perkin Elmer, Rodgau, Germany), and values were averaged after subtracting nonspecific binding. A standard curve was generated using a four-parameter logistic fit.

### 2.7. Mechanical Hyperalgesia Testing

Changes in mechanical hyperalgesia resulting from FCA-induced hindpaw inflammation was evaluated in animals (*n* = 5–6) with and without i.th. FAD286 delivery using a mechanical pressure device (Ugo-Basile SRL, Monvalle, Italy) as described previously [7,12]. Paw pressure thresholds (PPTs) that triggered a withdrawal response were recorded for all groups on the fourth day of FCA inflammation. Then, PPT measurements were repeated 2 h after the final intrathecal drug administration to assess the pharmacological effects. Throughout all behavioral testing, drug preparation was conducted by a separate individual (M.Sh.), while the experimenter (HoX) remained blinded to treatment allocation.

### 2.8. Statistics

All statistical analyses were performed using Sigma Stat 2.03 software (IBM SPSS Statistics, Ehningen, Germany). Algesiometry, qRT-PCR and ELISA results were expressed as means ± standard deviation (SD), tested for normality (Kolmogorov–Smirnov), and analysed using Student’s *t*-test. All statistical tests were two-sided, with an alpha level of <0.05, and were considered exploratory in nature.

## 3. Results

### 3.1. mRNA Transcript Detection of MR Together with Its Protecting Enzyme 11ß-HSD2 and Its Endogenous Ligand-Processing Enzyme CYP11B2 in Human Sensory DRG Neurons

**Figure 1:**
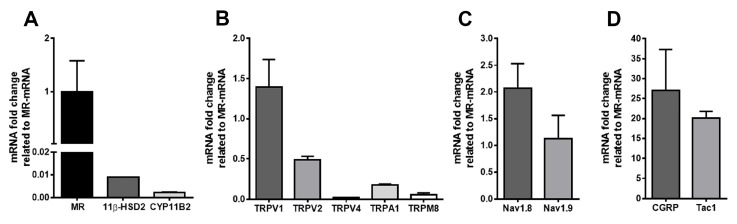
Detection of mineralocorticoid receptor (MR), protecting enzyme 11ß-HSD2, processing enzyme of endogenous ligand aldosterone CYP11B2 (**A**), and key pain signalling molecule (**B**–**D**) mRNA transcripts in human sensory DRG neurons. In using specific mRNA primers, quantitative real-time PCR confirmed the presence of mRNA transcripts for MR, 11β-HSD2, and CYP11B2 in human DRG tissue, with Ct values of 23, 30, and 32, respectively (**A**). In addition, quantitative real-time PCR analyses of human DRG neurons revealed that the mRNA expression levels of TRPV1 and Nav1.9 were approximately comparable to that of MR, whereas those of the other pain transduction receptors were markedly lower (**B**). (**C**,**D**) The mRNA expression levels of the TTX-resistant sodium channel Nav1.8, the neuropeptide CGRP, and substance P (Tac1), however, were approximately 2-, 20-, and 25-fold higher than that of the MR, respectively.

Using highly specific primer pairs (see Table S1), mRNA transcripts for the MR and its protective enzyme 11ß-HSD2 were detected in human DRG by qRT-PCR (Figure 1A). Given that neuronal MR are activated by their endogenous ligand aldosterone in rats [12], we confirmed the mRNA expression of the aldosterone-synthesizing enzyme CYP11B2 (aldosterone synthase) in human DRG (Figure 1A). Furthermore, we demonstrated the expression of pain-signalling molecules, previously shown to be genomically regulated by MR [8], in human DRG, including the heat- and proton-sensitive transduction receptors, including TRPV1, the nociceptive-specific TTX-resistant sodium channel Nav1.8, and the well-known nociceptive neuropeptides CGRP and substance P (Tac1). The mRNA expression levels of TRPV1 and Nav1.9 were comparable to that of MR, whereas the expression levels of the other pain transduction receptors were markedly lower (Figure 1B). Additionally, the mRNA expression levels of the TTX-resistant sodium channel Nav1.8, the neuropeptide CGRP and the substance P (Tac1) were approximately 2-, 20-, and 25-fold higher than MR, respectively (Figure 1C,D).

### 3.2. MR Receptor Detection in CGRP-IR Sensory Neurons Colocalizing with Its Protecting Enzyme 11ß-HSD2 in Human and Rat DRG Neurons

**Figure 2:**
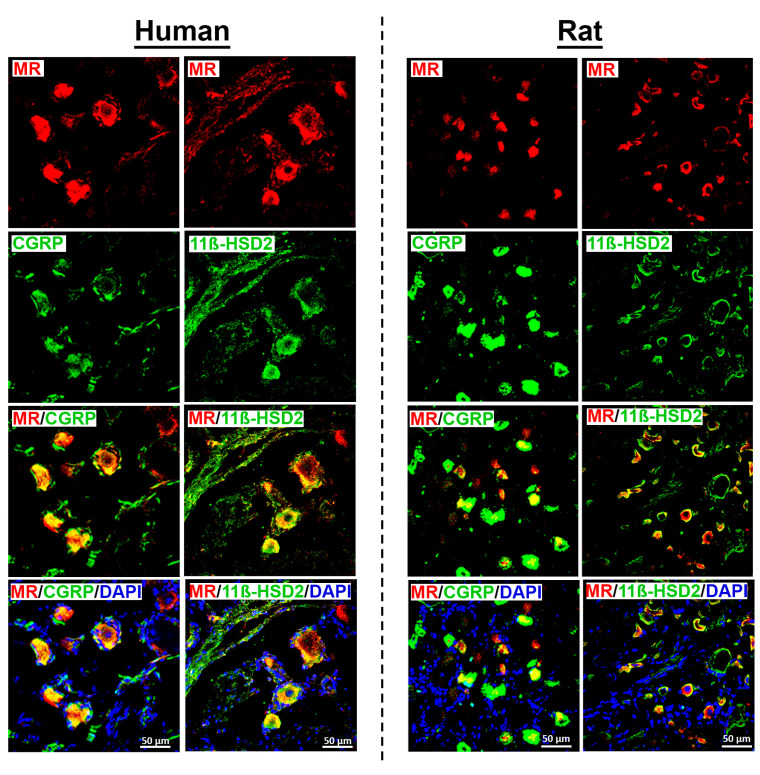
Detection of the mineralocorticoid receptors (MRs) with the peripheral sensory neuron marker CGRP and its protecting enzyme 11ß-HSD2 in human (left panel) and rat (right panel) sensory DRG neurons. MRs were visualized by a specific antibody (see Supplementary Table S2) and a respective secondary antibody labelled with red fluorescence, while CGRP was visualized by green fluorescence. Note, not all CGRP-IR neurons exhibited MR immunoreactivity, and conversely, some MR-IR neurons lacked CGRP immunoreactivity. MR and 11ß-HSD2 were visualized by specific antibodies (see Supplementary Table S2). The MR was labelled with red fluorescence, while the protecting enzyme 11ß-HSD2 was visualized by green fluorescence in both human (**left panel**) and rat (**right panel**) DRG neurons. Bars represent 50 µm.

Using well-characterized antibodies specific to human [10,18] and rat [12] mineralocorticoid receptors (MRs), we detected MR immunoreactivity in dorsal root ganglion (DRG) neurons from both species (Figure 2). Notably, rat DRG neurons appeared generally smaller than human DRG neurons when observed under the same magnification (Figure 2). MR immunoreactivity was predominantly localized in a subpopulation of peripheral CGRP-IR sensory neurons (Figure 2). However, not all CGRP-IR neurons exhibited MR immunoreactivity, and conversely, some MR-IR neurons did not express CGRP.

**Figure 3:**
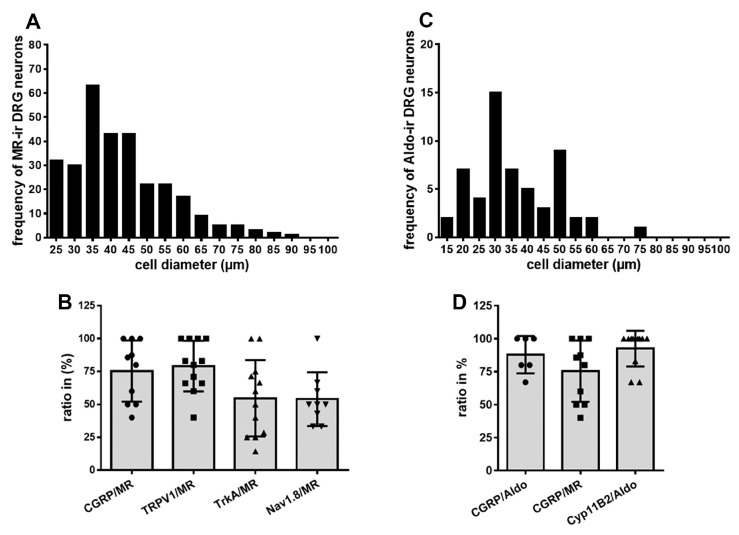
Semi-quantitative analysis of DRG neuron diameter and co-expression of MR (**A**,**B**) or aldosterone (**C**,**D**) with pain-related markers (CGRP, TRPV1, TrkA1, Na1.8) and CYP11B2 in human DRG neurons. (**A**,**C**) prominent subset of small-to-medium-diameter neurons (20–60 µm) demonstrated expression of MR (**A**) and aldosterone (**C**) in human DRG neurons consistent with a nociceptive profile. In contrast, a distinct population of large-diameter neurons (≥70 µm) exhibited much less likely MR and aldosterone. (**B**) Preliminary quantification of human DRG tissue samples showed that approximately 75% of MR-IR neurons were also CGRP-IR, and 43% were 11ß-HSD2-IR, suggesting that the MR in human DRG neurons may be partially protected from circulating glucocorticoids. (**D**) approximately 87% of aldosterone-IR neurons also expressed CGRP and 92% co-expressed CYP11B2; values are means ± SD.

MR-IR was primarily observed in small- to medium-diameter (25–60 µm) human DRG neurons, consistent with a nociceptive phenotype. In contrast, MR-IR was less frequently detected in large-diameter neurons (≥70 µm) (Figure 3A). Double immunofluorescence confocal microscopy revealed colocalization of MR with its protective enzyme 11ß-HSD2, which is also expressed in peripheral CGRP-IR sensory neurons, in both human and rat DRG (Figure 2). Preliminary quantification of human DRG tissue samples (*n* = 13–20) indicate that approximately 75% of MR-IR neurons were also CGRP-IR (Figure 3B), and 43% were 11ß-HSD2-IR, suggesting that the MR in human DRG neurons is at least partially protected from circulating glucocorticoids.

### 3.3. Identification of the MR Endogenous Ligand Aldosterone with Its Processing Enzyme CYP11B2 in Human and Rat DRG Neurons

**Figure 4:**
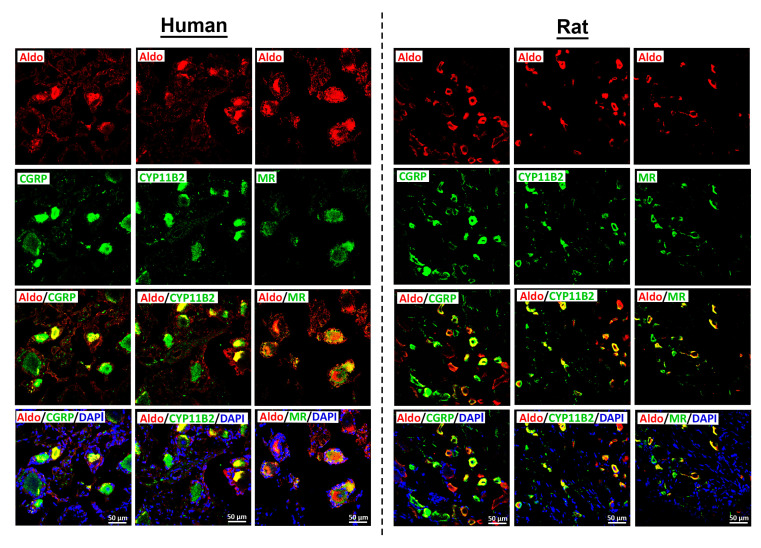
Detection of the endogenous aldosterone with the peripheral sensory neuron marker CGRP, its processing enzyme CYP11B2, and the mineralocorticoid receptors (MRs) in human (*n* = 2) versus rat (*n* = 5) sensory DRG neurons. Aldosterone was visualized by a specific antibody (see Supplementary Table S2), and respective secondary antibodies labeled with red fluorescence, while the CGRP, the processing enzyme CYP11B2, or the MR was visualized by green fluorescence. Note, the aldosterone immunoreactivity predominantly colocalizing with the sensory neuron marker CGRP, as well as with CYP11B2, in subsets of both human (**left panel**) and rat (**right panel**) DRG neurons. Bars represent 50 µm.

Most notably, we sought to identify not only the endogenous MR ligand aldosterone, but also its biosynthetic enzyme, aldosterone synthase (CYP11B2). Double immunofluorescence confocal microscopy revealed specific aldosterone immunoreactivity colocalizing with the sensory neuron marker CGRP, as well as with CYP11B2, in both human and rat DRG neurons (Figure 3C and Figure 4). Aldosterone and MR immunoreactivity colocalized with CGRP in 78% and 75% of cases, respectively (Figure 3D and Figure 4). Like MR-IR, aldosterone immunoreactivity (aldosterone-IR) was primarily localized to small- to medium-diameter (25–60 µm) human DRG neurons, consistent with a nociceptive phenotype, and was less frequently observed in large-diameter neurons (≥70 µm) (Figure 3C and Figure 4). Preliminary quantification of human DRG tissue samples indicate that approximately 78% of aldosterone-IR neurons also expressed CGRP, and 90% co-expressed CYP11B2 (Figure 3D).

### 3.4. Colocalization of MR with Key Pain-Signalling Molecules in Human and Rat DRG Neurons Exhibiting a Nociceptive Phenotype

**Figure 5:**
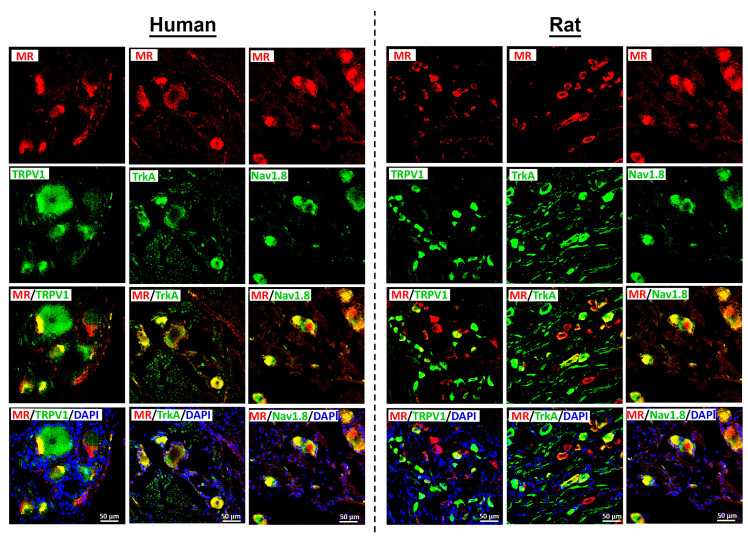
Detection of the mineralocorticoid receptors (MR) with the nociceptive-specific heat-sensitive TRPV1, neurotrophic tyrosine kinase receptor 1 (TrkA1) and the TTX-resistant voltage-gated sodium channel Nav1.8 in human (*n* = 2) versus rat (*n* = 5) sensory DRG neurons. MR is labelled with red fluorescence, while Nav1.8, TRPV1, TRKA1 are visualized by green fluorescence. Note, MR-IR neurons showed substantial colocalization with key pain-signaling molecules, including TRPV1, Nav1.8, and TrkA both in human (**left panel**) as well as in rat (**right panel**) DRG neurons. Bars represent 50 µm.

Since we previously demonstrated that the expression of key pain-signaling molecules is genomically regulated by the MR [8], we next investigated the co-expression of the MR with the TRP ion channel TRPV1, the TTX-resistant voltage-gated sodium channel Nav1.8, and the nerve growth factor (NGF) receptor TrkA in human DRG neurons. MR-IR neurons showed substantial colocalization with these key pain-signaling molecules, including TRPV1, Nav1.8, and TrkA (Figure 5). Preliminary quantification of human DRG tissue samples revealed that approximately 75% of MR-IR neurons also expressed TRPV1, 64% co-expressed Nav1.8, and 60% co-expressed TrkA (Figure 3B).

### 3.5. Chronic Intrathecal Inhibition of Aldosterone Synthase Prevents Both the Upregulation of Pain-Related Signaling Molecules and the Development of Mechanical Hypersensitivity During Inflammatory Pain

**Figure 6:**
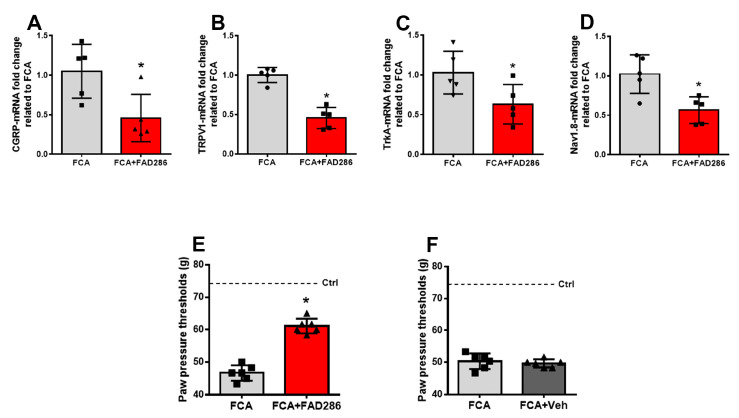
Continuous intrathecal aldosterone synthase inhibition reduced the expression of pain-related signaling molecules CGRP (**A**), TRPV1 (**B**), TrkA (**C**), and Nav1.8 (**D**) mRNA in rat dorsal root ganglia (DRG) and alleviated inflammation-induced mechanical hyperalgesia (**E**,**F**). Specifically, continuous intrathecal (i.t.) administration of FAD286 in rats with FCA inflammation significantly reduced the mRNA levels of CGRP (**A**), TRPV1 (**B**), TrkA (**C**), and Nav1.8 (**D**) (* *p* < 0.05, two-tailed independent Student’s *t*-test; *n* = 5). In panels (**E**,**F**), continuous i.t. infusion of FAD286 led to a marked increase in paw pressure thresholds (PPT) in contrast to i.t. vehicle treatment, demonstrating a reversal of FCA-induced mechanical hyperalgesia (* *p* < 0.05; *n* = 6 rats). PPT values of naïve rats are shown by the discontinuous line. Results are presented as means ± standard deviation (SD).

Given that genetic deletion, pharmacological inhibition, or overexpression of key pain-signaling molecules are directly associated with altered nociceptive thresholds and pain behaviors [19,20,21,22], we aimed to determine whether the expression of these molecules is regulated genomically by mineralocorticoid receptors (MRs) in peripheral neurons, and whether this regulation affects nociceptive processing. Using a rat model of unilateral inflammatory pain induced by hindpaw injection [23], we administered continuous intrathecal (i.t.) infusion of the aldosterone synthase inhibitor FAD286 (5 µL/h, 0.3 µg/µL). This treatment led to a significant 59% reduction in aldosterone content within the DRG (FCA + Vehicle: 7.4 ± 1.2 pg/mg; FCA + FAD286: 4.4 ± 0.8 pg/mg), along with a marked downregulation of inflammation-induced upregulation of CGRP, TRPV1, TrkA, and Nav1.8 mRNA expression levels (Figure 6A–D). These molecular changes were accompanied by a significant reversal of hyperalgesia (*p* < 0.05) (Figure 6E,F).

## 4. Discussion

In lumbar DRG neurons from two young individuals who died in fatal accidents, mRNA transcripts for the MR, its protective enzyme 11β-HSD2, and the aldosterone-synthesizing enzyme CYP11B2 were detected, with Ct values of 23, 30, and 32, respectively. These transcripts were identified alongside mRNAs encoding thermosensitive and pain-transducing receptors TRPV1, TRPV2, and TRPV4 (heat-/proton-sensitive); TRPA1 and TRPM8 (cold-sensitive); as well as the TTX-resistant sodium channels Nav1.8 and Nav1.9; and the nociceptive neuropeptides CGRP and substance P (Tac1). Consistently, immunoreactivity for the MR protein was detected in human DRG neurons using a validated MR-specific antibody previously applied to human tissue [18]. MR-IR human DRG neurons colocalized with 11β-HSD2, its protective enzyme, and with CGRP, a marker for sensory neurons of unmyelinated C- and thinly myelinated Aδ-fibers. Notably, MR-IR was predominantly observed in small- to medium-sized neurons (25–60 µm), with lower expression in large-diameter neurons (>70 µm). No MR immunoreactivity was detected in satellite glial cells or Schwann cells in either human or rat DRG.

These findings are consistent with previous studies in rats [5,6] and mice [24], which reported that MR-IR neurons are predominantly small to medium in size and frequently co-express CGRP (71%) and TrkA (56%), with a smaller proportion expressing NF200 (21%), a marker of myelinated neurons [6]. However, our results differ somewhat from a more recent study in both humans and mice employing RNAScope in situ hybridization, which demonstrated MR transcript expression in nearly all neurons (95–99%) [24]. This study also reported a positive correlation between MR mRNA puncta density and DRG neuron diameter, as well as a shift in the MR/GR (glucocorticoid receptor) mRNA puncta ratio favoring the MR in larger neurons [24]. These discrepancies may reflect differences between mRNA and protein expression levels. Our findings are also in line with earlier rat studies demonstrating behavioral and electrophysiological evidence for a pain-modulating role of MR in DRG neurons [5,6]. Specifically, local incubation of acutely dissociated DRG neurons with aldosterone (with a peak effect at 10 nM) resulted in an increased number of action potentials in response to suprathreshold current injections [5]. Furthermore, topical application of the MR antagonist eplerenone reversed the increase in evoked action potentials observed during zymosan-induced inflammation. These observations led to the authors’ presumption that MR activation in rat DRG neurons exerts an excitatory, pronociceptive effect [5], presumably through the endogenous ligand aldosterone [12].

Most interestingly, aldosterone was detected by immunohistochemistry in human DRG neurons, where it co-localized with the sensory neuron marker CGRP, its cognate receptor MR, and the cytochrome P450 enzyme CYP11B2, which catalyzes the final step in aldosterone biosynthesis, converting 18-hydroxycorticosterone to aldosterone [25]. Recent reviews [26,27] highlight accumulating evidence that aldosterone is synthesized in extra-adrenal tissues, including the heart and brain, where it exerts paracrine effects through MR signaling. Complementing these findings, earlier studies in rats have demonstrated CYP11B2 expression in DRG neurons at both the mRNA and protein levels [12]. Furthermore, CYP11B2 expression in sensory neurons appears to be upregulated under conditions of inflammatory pain [12].

Consistent with previous findings in rats [8], we now demonstrate that human DRG neurons also exhibit abundant co-localization of the mineralocorticoid receptor (MR) with key pain-related signaling molecules, including CGRP (75%), TRPV1 (76%), TrkA (56%), and Nav1.8 (55%). Given that MR is a nuclear hormone receptor known to regulate gene transcription and increasing evidence that, e.g., CGRP and TRPV1 gene transcription are genomically regulated by steroids [28,29], we sought to provide functional evidence supporting its role in nociception. In rats, chronic intrathecal inhibition of aldosterone synthase (CYP11B2) led to a 60% reduction in spinal aldosterone levels, accompanied by a significant downregulation of mRNA transcripts encoding key pain-signaling molecules. This molecular change was associated with a marked reversal of inflammation-induced hyperalgesia. These findings suggest that inhibiting aldosterone synthesis reduces both pain-signaling molecules and behavioral signs of hyperalgesia, underscoring the potential of aldosterone and MR as therapeutic targets for pain modulation. Furthermore, this finding aligns with previous studies [28,29], which demonstrated that activation of MR on sensory DRG neurons modulates the expression of neuronal pain-signaling molecules, such as TRPV1.

Our study has some limitations. Most notably, the human data are based on only two DRG samples (one male, one female), which limits generalizability. Although we used well-validated antibodies and performed representative control experiments, the possibility of antibody cross-reactivity cannot be entirely excluded. Nevertheless, our immunohistochemical results were corroborated by transcriptional data obtained through qRT-PCR. To strengthen these findings, a more comprehensive investigation of MR and aldosterone distribution, including the enzymes involved in its biosynthesis, should be conducted in a larger and more diverse cohort of human DRG samples.

## 5. Conclusions

In conclusion, the MR system is expressed in human DRG neurons and co-localizes with key pain-related molecules. Through transcript profiling and immunofluorescence analysis, we provide evidence linking the MR to nociceptive signaling pathways. Furthermore, functional inhibition of aldosterone synthesis in rats attenuated pain markers and hyperalgesia, reinforcing the therapeutic potential of targeting MR in pain management. While MR is traditionally recognized for its role in regulating sodium and water homeostasis via aldosterone-responsive gene expression in the kidney [30], our findings suggest it may also play a crucial role in modulating nociceptive processes in humans.

## Data Availability

Data are available upon reasonable request by contacting the first author at shaaban.mousa@charite.de.

## Supplementary Materials

The following supporting information can be downloaded at: https://www.mdpi.com/article/10.3390/cells14151142/s1, Figure S1: Immunofluorescence staining of human dorsal root ganglia tissue using Alexa Fluor 594 donkey anti-rabbit antibody (Texas red immunofluorescence) and Alexa Fluor 488 goat anti-mouse antibody (FITC green fluorescence) as secondary antibodies with omission of the respective primary antibodies (blank control); Table S1: List of Primers for Taqman RT-PCR of human DRG; Table S2: Characterization of primary antibodies used.
