# Supplementary material for: Components of Mineralocorticoid Receptor System in Human DRG Neurons Co-Expressing Pain-Signaling Molecules: Implications for Nociception
Source: Cells. 2025 Jul 24;14(15):1142. doi: 10.3390/cells14151142 (PMC12346002; doi:10.3390/cells14151142)

Table S1: List of Primers for Taqman RT-PCR of human DRG

Gene	Access. Nr.	Forward	Reverse
**MR**	NM_000901.4	5′-aaagagcagtggaagggcaa-3′	5′-tgaagtctgcaagcaggacaa-3′
**HSD11B2**	NM_000196.4	5′-ccaccagcaccggcct-3′	5′-cctccatgcagctacggaaa-3′
**CYP11B2**	NM_000498.3	5′- caaggagaccttgcggctctac-3′	5′-cccagcgagtagaggaaaacctg-3′
**TRPV1**	NM_080704.4	5′-gcaggacaagtgggacagat-3′	5′-tcttaaagggaggcaagcca-3′
**TRPV2**	NM_016113.5	5′-aaactgctgcaggcgaaatg-3′	5′-ggcggcctgcttcttca-3′
**TRPV4**	NM_021625.5	5′-tcaaagtcttcaaccggcct-3′	5′-ccccgtagatggctctcgaa-3′
**TRPA1**	NM_007332.3	5′-ggggccctggttctgtaaat-3′	5′-atacgcccataactggctgc-3′
**TRPM8**	NM_024080.5	5′-aaactggttgcgaacttccg-3′	5′-taggagacacgtcgtggagt-3′
**Nav.1.8**	NM_006514.4	5′ctccgtccttgaggaactcg-3	5′-tctgcaaagggatccgtcac-3′
**Nav.1.9**	NM_001349253.2	5′agccaggaatctcgggtgaa-3	5′-agccagagagtcggaagtga-3′
**CGRP**	NM_001378950.1	5′actggtgcaggactatgtgc-3	5′-actggtgcaggactatgtgc-3′
**NTRK1 (TrkA)**	NM_002529.4	5′cagagaacctgactgagctctac-3	5′-gaaatggaaggcatctggcg-3′
**S18**	NR_046237	5′-cggctaccacatccaaggaa-3′	5′-cctggaattaccgcggct-3′

Table S2: Characterization of primary antibodies used

Antigen	Immunogen	Manufacturer, Species, Type, Catalogue Number	Dilution used
**MR**	a 142-amino-acid peptide sequence from the unique DNA-binding domain of the rat MR gene	a gift from M. Kawata (Kyoto Prefectural University of Med Japan), rabbit polyclonal,	1:2.000
**MR**	generated against epitopes located at the N terminal of the MR molecule	Elise Gomez-Sanchez, Jackson, USA, rMR 79–87 monoclonal antibody [18]; [10]	1.200
**Aldosterone**	Aldosterone-3-CMO-BSA	(Novus Biologicals, LLC, CO, USA), rabbit polyclonal, # MB100-64658	1:2.000
**CYP11ß2**	Chicken serum albumin-conjugated linear peptide corresponding to human CYP11ß2 near the N-terminus	Millipore (Darmstadt, Germany), mouse monoclonal, # clone 41-17B Cat # MABS1251 [27]	1:1.000
**11ßHSD2**	raised against the recombinant rat 11ßHSD2 protein in sheep	Elise Gomez-Sanchez, Jackson, USA, sheep polyclonal	1:3.00
**11ßHSD2**	Recombinant fusion protein containing a sequence corresponding to amino acids 266-405 of human HSD11B2	St. John's Laboratory Ltd., London, UK. Rabbit polyclonal, (NP_000187.3).	1:1000
**CGRP**	synthetic entire calcitonin gene-related peptide	Peninsula Laboratories (CA, USA), guinea pig polyclonal, # T-5027 [13]	1:1.000
**TRPV1**	VR1 C-terminus (TRPV1)	Neuromics MN, USA), guinea pig polyclonal # GP14100	1:1.000
**Nav1.8**	a peptide (C)EDEVAAKEGNSPGPQ corresponding to residues 1943-1957 of rat Nav1.8	Sigma-Aldrich (USA), polyclonal rabbit, # S2071 [8]	1:1.000
**trkA**	extracellular domain Ala33-Pro418 of rat trkA	R&D Systems (USA), goat polyclonal, # AF1056	1:1,000

Reference:

8. Shaqura, M.; Li, L.; Mohamed, D.M.; Li, X.; Treskatsch, S.; Buhrmann, C.; Shakibaei, M.; Beyer, A.; Mousa, S.A.; Schäfer, M. Neuronal aldosterone elicits a distinct genomic response in pain signaling molecules contributing to inflammatory pain. *Journal of neuroinflammation* **2020**, 17, 183, doi:10.1186/s12974-020-01864-8.
10. Tafelski, S.; Wandrey, J.D.; Shaqura, M.; Hong, X.; Beyer, A.; Schäfer, M.; Mousa, S.A. Translation of Experimental Findings from Animal to Human Biology: Identification of Neuronal Mineralocorticoid and Glucocorticoid Receptors in a Sectioned Main Nerve Trunk of the Leg. *Cells* **2023**, 12, doi:10.3390/cells12131785.
13 Mousa, S.A.; Shaqura, M.; Tafelski, S.; Wandrey, J.D.; Celik, Ö.; Treskatsch, S.; Schäfer, M. The Identification of Opioid Receptors and Peptide Precursors in Human DRG Neurons Expressing Pain-Signaling Molecules Confirms Their Potential as Analgesic Targets. *Cells* **2025**, 14, doi:10.3390/cells14100694.
18. Ibarrola, J.; Lopez-Andres, N. Editorial: Heart valve diseases: from molecular mechanisms to clinical implications. *Front Mol Med* **2023**, 3, 1260912, doi:10.3389/fmmed.2023.1260912.
27. Gomez-Sanchez, C.E.; Qi, X.; Velarde-Miranda, C.; Plonczynski, M.W.; Parker, C.R.; Rainey, W.; Satoh, F.; Maekawa, T.; Nakamura, Y.; Sasano, H.; et al. Development of monoclonal antibodies against human CYP11B1 and CYP11B2. *Molecular and cellular endocrinology* **2014**, 383, 111-117, doi:10.1016/j.mce.2013.11.022.

Supplemental Figure 1:

Immunofluorescence staining of human dorsal root ganglia tissue using Alexa Fluor 594 donkey anti-rabbit antibody (Texas red immunofluorescence) and Alexa Fluor 488 goat anti-mouse antibody (FITC green fluorescence) as secondary antibodies with omission of the respective primary antibodies (blank control). Nuclei were counterstained with 4′,6-diamidino-2-phenylindole (DAPI; bright blue). Scale bar = 40 μm.

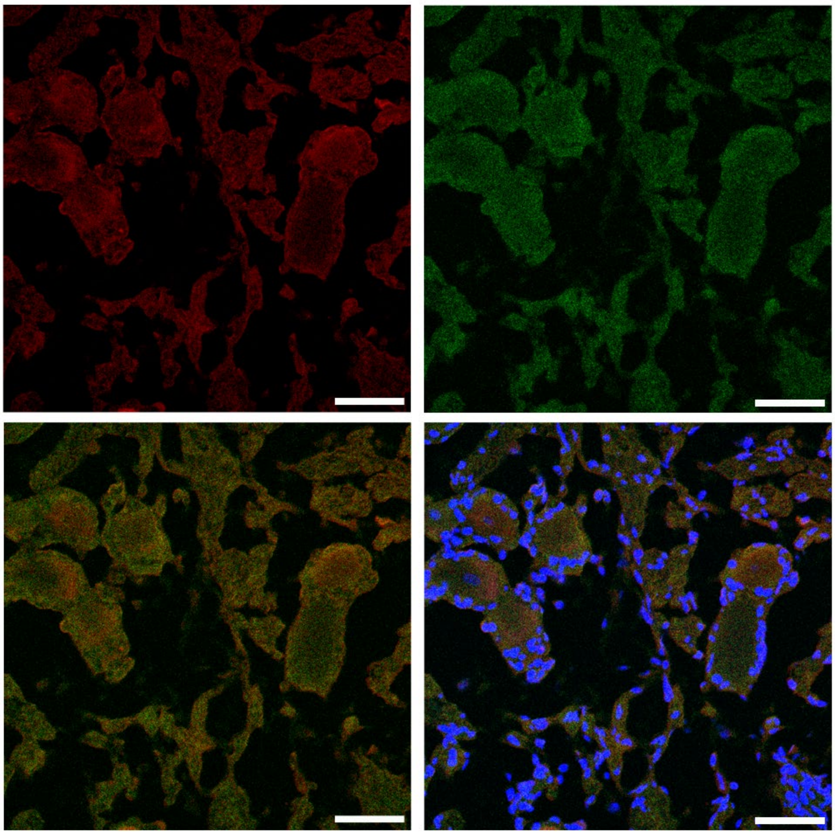

Supplement: Supplementary file 1 [file cells-14-01142-s001.zip › cells-3763460-supplementary.pdf]
